# Lack of evidence of mastitis as a causal factor for postpartum dysgalactia syndrome in sows^1,2,3^

**DOI:** 10.1093/tas/txz159

**Published:** 2019-10-11

**Authors:** Marianne Kaiser, Magdalena Jacobson, Poul Bækbo, Jan Dahl, Stine Jacobsen, Yong Z Guo, Torben Larsen, Pia H Andersen

**Affiliations:** 1 Department of Veterinary Clinical Sciences, Copenhagen University, Taastrup, Denmark; 2 Department of Clinical Sciences, Swedish University of Agricultural Sciences, Uppsala, Sweden; 3 SEGES, Danish Pig Research Centre, Aarhus N, Denmark; 4 Danish Agriculture and Food Council, Axelborg, Copenhagen V, Denmark; 5 Department of Animal Science, Aarhus University, Tjele, Denmark

**Keywords:** bacteria, clinical diagnosis, lipopolysaccharide, milk, PDS, sows

## Abstract

To investigate the prevalence of mastitis in sows suffering from postpartum dysgalactia syndrome (**PDS**), we examined milk constituents and concentrations of lipopolysaccharides (**LPS**) obtained from the udder vein (v. epigastrica). As part of a case–cohort study, 109 sows were monitored daily from 60 h antepartum (**a.p.**) to 36 h postpartum (**p.p.**). Over time, 38 sows were diagnosed with PDS (PDS+) and were retrospectively matched with 38 healthy sows (PDS−). The study period was divided into 7 smaller time periods (A, B, C, D, E, F, G, H, and E), allowing the studied values, in period B to G, to be compared with period A that served as a baseline, and PDS+ and PDS− sows were compared within the time periods. All sows were subjected to a thorough daily clinical examination and blood was sampled from v. epigastrica for LPS detection. Milk samples were obtained for bacteriological evaluation and detection of *N*-acetyl-beta-d-glucosaminidase (**NAGase**), lactate dehydrogenase (**LDH**), β-glucuronidase (**β-glu**; for evaluation of mastitis), isocitrate (**isoC**), free glucose, uric acid (**UA**; for evaluation of the mammary energy status), β-hydroxybutyrate acid (**BHBA**; for evaluation of ketosis), and milk urea (for evaluation of the protein status). The results revealed that PDS+ sows had decreased concentrations of UA in milk (*P* < 0.0001), increased heart rates (*P* < 0.01), increased mammary edema (*P <* 0.05), and prolonged capillary refill time in the vulvar mucosa (*P* < 0.01) compared with PDS− sows. Compared with baseline, feces became more solid 0 to 36 h p.p. (*P* < 0.0001) and the respiration rate decreased 12 to 24 h p.p. (*P* < 0.0001) for both PDS+ and PDS− sows. No differences were found between PDS+ and PDS− sows for severe bacterial infections, concentrations of LPS in blood or LDH, NAGase, BHBA, free glucose, isoC, or urea in milk. Concentrations of LPS in blood were not associated with signs of mastitis or edema in the mammary glands. However, a difference over time was seen for redness (*P* < 0.0001), warmth (*P* < 0.0001), and hardness (*P* < 0.05) of the 6 most anterior glands in both PDS+ and PDS− sows from 60 h a.p to 36 h p.p. The PDS− sows had greater concentrations of β-glu than the PDS+ sows, but no change over time was demonstrated for this marker. In conclusion, signs of mastitis were not consistently linked to PDS in sows. However, the cardiovascular system seemed to be compromised in PDS+ sows and the cause should be investigated to elucidate the pathogenesis of PDS.

## INTRODUCTION

Postpartum dysgalactia syndrome (**PDS**) in sows has a complex multifactorial pathophysiology (Martineau et al., 2013). Risk factors include nutrition, housing, management (Papadopoulos et al., 2010), and genetic (Preissler et al., 2012). Further, the diagnostic values of clinical signs are unclear. Infectious mastitis can cause agalactia (Gerjets and Kemper, 2009), mainly due to *Escherichia coli* (*E. coli*) (Persson et al., 1996; Gerjets and Kemper, 2009). *Escherichia coli* and its derived cell membrane components lipopolysaccharides (**LPS**) can induce inflammatory changes and signs of mastitis after intramammary challenge in sows (Zhu et al., 2004; Wang et al., 2006). Lipopolysaccharides were identified in mammary blood of cows with coliform mastitis (Katholm and Andersen, 1992), yet this has not been studied in sows.

Markers for mastitis have not been studied extensively in sows when compared with cows, but it is conceivable that such markers may also be useful in sows. Bovine milk *N*-acetyl-beta-d-glucosaminidase (**NAGase**) and lactate dehydrogenase (**LDH**) were shown to be positively correlated with inflammatory markers (Larsen et al., 2010), and β-glucuronidase (**β-glu**), LDH, and NAGase are commonly used as markers of bovine mastitis (Larsen and Aulrich, 2012). In addition, isocitrate (**isoC**) (Larsen, 2014), free glucose (Larsen, 2015), and uric acid (**UA**) can also be used for a comprehensive health assessment of cows (Larsen and Moyes, 2010) as they assess the mammary energy status. Finally, milk β-hydroxybutyrate acid (**BHBA**) (Larsen, 2015) and milk urea are related to metabolic status (ketosis and protein balance, respectively) in cows (Nielsen et al., 2005). Use of these various markers could prove useful in the search for simple milk tests for research purposes.

The aim of this study was to investigate the development of mastitis in sows with PDS (PDS+) or healthy sows (PDS−) using clinical examination, milk bacteriology, analyses of milk constituents, and LPS concentrations in blood. It was hypothesized that mastitis would be more prevalent in PDS+ sows, and that changes in milk constituents, and concentrations of LPS in blood could serve as markers of mastitis in sows.

## MATERIALS AND METHODS

### Definition of PDS

The experimental protocol was approved by the Danish Animal Experiments Inspectorate (permit: 2013-15-2934-00970). Sick animals were given medical treatment in accordance with guidelines on animal welfare and ethical accountability. We therefore applied a rather broad clinical diagnosis of PDS, where at least 2 of the following 3 clinical characteristics had to be presence: 1) anorexia (trough not empty 30 min after feeding), 2) mastitis (redness, swelling, and increased skin temperature), and 3) high rectal temperature ≥ 39.5 °C.

### Study Design

A total of 109 multiparous sows were monitored in a case–cohort study, resulting in the selection of 38 PDS+ and 38 PDS− sows in the periparturient period. The 109 sows were randomly selected from 9 different batches entering the farrowing unit. All sows were examined and sampled every 24 h from 60 h antepartum (**a.p.**) to 36 h postpartum (**p.p.**). Sows diagnosed as PDS+ sows were continually treated and then withdrawn from the study.

After data collection, PDS+ sows were retrospectively matched with PDS− sows according to: 1) batch, 2) parity, and 3) date of farrowing, in respective order of importance. In total, 76 individuals were included in the final data analyses (38 PDS+, 38 PDS−).

The trial was conducted in 1 herd of Danish cross-breed (Landrace/Yorkshire) pigs within the Danish Specific Pathogen-Free system. From 1 wk a.p. until 3 wk p.p., sows were housed in farrowing pens measuring 1.6 × 2.6 m^2^ with partly slatted floors (2/3 solid concrete; 1/3 iron bars). The pens were cleaned and disinfected (Bayocide, Bayer A/S, Lyngby, Denmark) between batches. The lactation feed was given throughout the study period and consisted of home-mixed liquid feed (50% barley and 50% wheat). The sows were fed restrictively with approximately 2.7 kg/d from 2 d before expected farrowing. This feed amount was increased with approximately 0.9 kg daily after farrowing until 8 or 9 d later. Straw were allocated according to the Danish animal welfare regulations that gives the sows a sufficient amount of straw to meet their need for rooting, satiety, and chewing (Bekendtgørelse af lov om indendørs hold af smågrise, avls- og slagtesvin, Kapitel 2, § 5, https://www.retsinformation.dk/Forms/R0710.aspx?id=186232). The occurrence of PDS, 20% to 30% of all sows at the farm, was estimated from the treatment frequency in the periparturient period.

### Experimental Protocol

Before farrowing, a daily monitoring and sampling were performed in the morning hours after feeding. The monitoring consisted of a thorough clinical evaluation and assessment of each mammary gland according to the definitions listed in Supplementary Appendix 1, and a blood sampling from v. epigastrica cranials superficialis (v. epigastrica) as described below. After farrowing, the monitoring was followed by milking immediately after farrowing and a second milking when the sows left the study (the procedure is described in detail below).

When sows were diagnosed as PDS+, they were immediately sampled, treated, and withdrawn from the study. Medical treatment included either 10,000 IU/kg BW of benzyl procaine penicillin (Noropen vet., ScanVet, Fredensborg, Denmark) or 16 mg/kg BW of trimethoprim-sulfadiazine (Norodine vet., ScanVet, Fredensborg, Denmark), and 0.4 mg/kg BW of meloxicam (Loxicom, ScanVet, Fredensborg, Denmark). Sows that farrowed too early to provide adequate samples and those treated for reasons other than PDS were excluded from the study.

### Sampling of Blood for LPS Determination

The v. epigastrica was located by palpation of the furrow at the transition between the udder and the sow’s abdomen—above the second nipple. Gross contamination on the udder was previously removed using a sponge and warm water with liquid soap (Neutral, Unilever Danmark A/S, Copenhagen S, Denmark) and the skin was disinfected (5% chlorhexidine gluconate, Jørgen Kruuse A/S, Langeskov, Denmark) using sterile swabs and sterile gloves. The blood was collected in 1.5-mL vacutainer tubes containing sodium citrate (BD, Franklin Lakes, NJ). The tubes were stored on ice within 30 min of sampling, wiped with disinfectant (5% chlorhexidine gluconate, Jørgen Kruuse A/S, Langeskov, Denmark), and centrifuged at 1,000 × *g* for 30 min at room temperature. The plasma was pipetted into pyrogen-free test tubes (Lonza, Basel, Switzerland) using sterile syringes, needles, and gloves. Plasma was frozen at −18 °C and stored for a maximum of 10 d, followed by storage at −80 °C until transportation on dry ice to the laboratory for analysis. The LPS concentration was tested by the commercially available kit PyroGene Recombinant Factor C no. 50-658U (Lonza, Walkersville, MD). Briefly, all samples were vigorously vortexed and diluted with Limulus Amebocyte Lysate Reagent Water (1:80; Lonza, Walkersville, MD). After being heated at 70 °C for 25 min, samples were cooled to room temperature. Then 100 µL of endotoxin standards, blanks, and diluted samples were added into the appropriate wells of the microplate and the plates were preincubated at 37 °C for 10 min. Following this, 100 µL of working reagent was added to each well and read immediately. The excitation filters were λ = 380 nm and the emission filter was λ = 440 nm; optics position for reading the well is from the bottom of the plate. The plates were read again 1 h later. All standards and samples were tested in duplicate. A FLx800 LBS Fluorescence Reader (BioTek Instruments, Winooski, VT) and WinKQCL 5 (Lonza, Walkersville, MD), that automatically calculates the amount of endotoxin recovered in the Positive Product Control, allowing a comparison with the known amount of endotoxin spike, were used in this assay. All standards and samples were tested in duplicate using multiple assay plates, and the average of the individual coefficients of variation (CVs) reported here as the intra-assay CV (≤15%).

### Sampling of Milk

Milking of 2 milk samples (no. 1 and no. 2) were obtained from each sow on 2 occasions; one milking within approximately 6 h after farrowing and a second milking when the sows exited the study (day 1 or 2). Administration of 2 mL oxytocin (10 IU/mL) per sow (Oxytocin “Intervet” Vet., MSD Animal Health, Copenhagen V, Denmark) was permitted at the second milking to enhance the milk ejection, as oxytocin would have no influence on the study at this time point.

Milk was preferably obtained from glands with signs of mastitis, but if there were no signs of inflammation in any gland, 1 of the 2 most anterior glands was chosen for milking. If milking was impossible or the quantity of milk obtained was inadequate at the first milking, the procedure was repeated on the gland closest to the first selected gland. To avoid contaminated milk, any milk present in the nipples (ductus papillaris) was removed before sampling, followed by thorough disinfection (5% chlorhexidine gluconate, Jørgen Kruuse A/S, Langeskov, Denmark) with sterile swabs and sterile gloves before the milk sample was obtained.

Milk sample no. 1 required sterile and few drops were used for bacteriological analysis. After milking, the sample was immediately collected with a 10-μm loop and spread on Colombia blood agar (CM033, Oxoid A/S, Roskilde, Denmark) in a clean room next to the barn. The agar plates were kept at 37 °C for 24 h and thereafter stored bottom-up at −5 °C for a maximum of 4 d before being manually read at the Laboratory of Swine Diseases (SEGES, Kjellerup, Denmark). A quantitative count of bacterial species was performed for each plate by a trained employee at the laboratory. In case of an abundance of bacterial colonies, the samples were serially diluted and re-analyzed. Substantial bacterial infections were defined as >10 cfu/10 µL.

Milk sample no. 2 (approx. 6 mL; unfiltered) was pipetted into 2-mL micro-tubes (SafeSeal, Sarstedt AG & Co., Nümbrecht, Germany) at the farm and stored as described for plasma until laboratory analyses were performed at Aarhus University, Dept. of Animal Science, Tjele, Denmark. Lactate-dehydrogenase activity (EC 1.1.1.27), β-glu activity (EC 3.2.1.31), NAGase activity (EC 3.2.1.30), and the metabolites BHBA, isoC, free glucose, UA, and urea were analyzed in plasma according to the following methods: LDH, β-glu, and NAGase activity were determined by fluorometry and kinetic assays (Larsen, 2005). β-Hydroxybutyrate acid (Larsen and Nielsen, 2005), isoC (Larsen, 2014), free glucose (Larsen, 2015), and UA (Larsen and Moyes, 2010) were measured by enzymatic-fluorometric assays. Milk urea was analyzed using flow injection analyses according to the manufacturer’s instructions (Foss Tecator AB, Höganäs, Sweden). Urease (EC 3.5.1.5) was added to the dilute milk sample; after the reaction, a strong alkali solution was added, and the developing ammonia was dialyzed through a membrane. pH changes in the passing, aqueous phase were followed by spectrophotometry by changes in color via a pH indicator (Nielsen et al., 2005). Measurements were not in duplicates in this instance but the laboratory reports that the CVs for all measurements (variables) normally were within 6% (intra-assay) and 7.5% (inter-assay).

### Statistical Analyses

Birth of the first piglet was recorded by video and precisely determined with the exact sampling times for each test value (date:hour:min). It was therefore possible to create a data management program (SAS Institute, Cary, NC) that related the exact sampling times to birth of the first piglet. Subsequently, the monitoring period (60 h a.p. to 36 h p.p.) was divided into the following 7 time periods, where 0 h represented the initiation of farrowing: A. −60 to −36 h; B. −36 to −24 h; C. −24 to −12 h; D. −12 to 0 h; E. 0 to 12 h; F. 12 to 24 h, and G. 24 to 36 h.

Clinical variables with observations ≤ 5 were not included in the statistical analysis. Capillary refill time was assessed at vulvar skin and skin of gland no. 4, normal pink vulvar mucosa, feces assessment by rectal examination, hard gland, and gland edema (Supplementary Appendix 1) were analyzed according to the following autoregressive logistic regression model in the PROC GENMOD procedure of Statistic Analytical Software Enterprise Guide 7.1 (SAS Institute, Cary, NC): OUTCOME PARAMETER_*ij*_ = μ + TIME_*i*_ + GROUP_*j*_ + TIME * GROUP_*ij*_ + Φμ _*t*−1_ + ε _*ij*_, where OUTCOME PARAMETER_*ij*_ was the class variables with only 2 categories, μ was the observation value at time 0, TIME_*i*_ was the explanatory variable “time periods A–G,” GROUP_*j*_ was the explanatory variable “PDS+/PDS−,” TIME * GROUP_*ij*_ was the interaction between the 2 groups and change over time, Φμ _t−1_ was the explanatory variables for the binary outcomes, and ε _*ij*_ was the random residual error term. If the TIME * GROUP_*ij*_ interaction was nonsignificant, model A was rejected.

Class variables with several ordered categories (fecal consistency; Supplementary Appendix 1) were transformed to a progression scale (e.g., 1, 2, 3) and analyzed by the generalized linear mixed model in the PROC GLIMMIX procedure of Statistic Analytical Software Enterprise Guide 7.1 (SAS Institute, Cary, NC), assuming a multinomial distribution and a cumulative logit link. Feces were categorized by the following numbers: very wet feces, unformed and liquid = 1, between normal and wet, still formed but not solid = 2, normal and soft, but solid and well-formed = 3, between dry and normal = 4, dry and pellet-shaped = 5, absence of feces = 6.

Numerical variables (heart rate, respiratory rate, β-glu, isoC, free glucose, UA, BHBA, urea, and LPS in v. epigastrica) were analyzed by the following 2 statistical models (A or B). Model A: OUTCOME PARAMETER_*ij*_ = μ + TIME_*i*_ + GROUP_*j*_ + TIME * GROUP_*ij*_ + ε _*ij*_, where OUTCOME PARAMETER_*ij*_ was the value of the nominal variables, μ was the observation value at time 0 (corresponding to the mean of time period A), TIME_*i*_ was the explanatory variable “time periods A–G,” GROUP_*j*_ was the explanatory variable “PDS+/PDS−,” TIME * GROUP_*ij*_ was the interaction between the 2 groups and change over time, and ε _*ij*_ was the random residual error term. Since an exact match on all sows was not possible in all instances, the sows were in reality not matched, except for the sampling time, the effect of batch number was not included in the model. Least squares means (LSMEANS) and standard deviations (SD) were included in model A. If the TIME * GROUP_*ij*_ interaction was nonsignificant, model A was rejected. In case of nonsignificant interaction, model A was rejected and replaced with statistical model B: OUTCOME PARAMETER_*ij*_ = μ + TIME_*i*_ + GROUP_*j*_ + ε _*ij*_. If a nonsignificant change in TIME_*i*_ occurred in model B, the OUTCOME PARAMETER_*ij*_ was considered nonsignificant. For significant TIME_*i*_ values, differences in the relevant groups were still accepted and recorded from the model A output. In case of a significant effect of GROUP_*j*_, an overall effect of group was accepted. Statistical significance was considered when *P* < 0.05. Parity and body condition score were included as explanatory variables. From the preliminary analyses, neither obstetric aid nor farrowing length was found to be associated with any of the outcome variables. The following variables were transformed using the natural logarithm in order to improve the normality of residuals: LPS in v. epigastrica, LDH, β-glu, isoC, free glucose, UA, BHBA, and respiratory rate. Standard deviations for these variables cannot be directly interpreted because they are backwards transformed and not normally distributed.

Associations between LPS in v. epigastrica and mammary clinical variables (redness, warmth, hardness, and edema) in the 6 most anterior glands, as well as the change over time in the appearance of these glands were calculated by the PROC GLIMMIX procedure of Statistic Analytical Software Enterprise Guide 7.1 (SAS Institute, Cary, NC).

It should be noted that the number of observations in the 7 intervals varied because the sampling times varied relative to individual farrowing (0 h).

## RESULTS

### General Clinical Appearance and Reproduction

Rectal temperatures, appetite, and signs of mastitis are summarized in Table 1. Notably, the mean temperature (39.0 °C) within PDS− sows were highest between 12 and 24 h p.p. ranging from 39.0 °C for the sow with the lowest temperature to 39.3 °C for the sow with the highest temperature. Numerically, it seems that red and warm glands occurred more often in PDS+ sows than PDS− sows after farrowing. Reduced appetite was observed in 10 PDS+ sows and 5 PDS− sows from 0 to 12 h p.p., and in 12 PDS+ sows and 12 PDS− sows in the following time period (12 to 24 h p.p.). Four PDS+ sows and 2 PDS− sows had reduced appetite from 24 to 36 h p.p. Ceased appetite was observed on 3 occasions in the PDS+ sows from 12 to 26 h p.p. (Table 1).

**Table 1.** Clinical signs

Variable	Group		A. (−60 to −36 h)	B. (−36 to −24 h)	C. (−24 to −12 h)	D. (−12 to 0 h)	E. (0 to 12 h)	F. (12 to 24 h)	G. (24 to 36 h)
Temperature, °C	PDS+	Mean	38.1	38.2	38.2	38.3	38.9	39.5	39.6
		Min.	37.4	37.4	37.9	37	38	38.1	39
		Max.	38.7	38.9	38.4	39.5	40.1	40.5	40.5
	PDS−	Mean	38.2	38.3	38.2	38.2	38.6	39	38.8
		Min.	37.6	37.6	37.8	37.2	37.3	38.1	38.1
		Max.	38.8	38.8	38.4	39.1	39.3	39.3	39.3
Red gland, number	PDS+	Median	0	0	0	0	4.5	9	14
		Min.	0	0	0	0	0	0	4
		Max.	15	17	14	17	16	17	14
	PDS−	Median	0	0	0	0	0	14	7
		Min.	0	0	0	0	0	0	0
		Max.	16	16	16	16	15	16	15
Warm gland, number	PDS+	Median	0	0	0	0	0	14	14
		Min.	0	0	0	0	0	0	1
		Max.	1	1	14	7	16	17	14
	PDS−	Median	0	0	0	0	0	3.5	0
		Min.	0	0	0	0	0	0	0
		Max.	14	16	14	7	15	16	15
Appetite, number	PDS+	Normal	11	14	5	14	5	9	2
		Reduced	6	9	5	9	10	12	4
		Ceased	0	0	0	0	0	1	2
	PDS−	Normal	14	17	9	20	5	13	12
		Reduced	8	11	3	7	5	12	2
		Ceased	0	0	0	0	0	0	0

Results of clinical signs monitored during 7 time slots (A–G, from −60 h antepartum until 36 h postpartum) in sows with (*n* = 38) and without (*n* = 38) postpartum dysgalactia syndrome (PDS+ and PDS−, respectively).

Observations of a dark red vulvar mucosa did not differ between the PDS+ and PDS− sows (*P* = 0.07; data not shown), but a significant increase of redness was demonstrated at −12 to 0 h a.p. (*P <* 0.05) and 0 to 12 h p.p. (*P* < 0.01) compared with baseline A (−60 to −36 h a.p.; data not shown). Several of the clinical variables were either not observed or were observed for a small number of sows (≤5) and were therefore not used for any further statistical analyses (Supplementary Appendix 1). Vulvar ulcers were observed in 23 sows. One sow was excluded because of a rectal prolapse and another due to a vaginal prolapse. The body condition of the sows was described in a previous publication (Kaiser et al., 2018b).

### Cardiovascular System and Respiration

An overall (−60 h a.p. to 36 h p.p.) difference between PDS+ and PDS− sows was found for heart rate (*P* < 0.01; Table 2). There were no differences between PDS+ and PDS− sows for respiratory frequency, but a significant decrease in frequency was seen for both groups at 0 to 36 h p.p. compared with the baseline (*P*-values are shown in Table 2). The capillary refill time of the vulvar mucosa was significantly prolonged in PDS+ sows compared with PDS− sows (*P* < 0.01; Table 3).

**Table 2.** Heart beats, respiratory rate, and concentration of lipopolysaccharide (LPS)

			A. (−60 to −36 h)		B. (−36 to −24 h)		C. (−24 to −12 h)		D. (−12 to 0 h)		E. (0 to 12 h)		F. (12 to 24 h)		G. (24 to 36 h)	
Variables	Group	*n*	LSMEANS	±SD	LSMEANS	±SD	LSMEANS	±SD	LSMEANS	±SD	LSMEANS	±SD	LSMEANS	±SD	LSMEANS	±SD
Heart rate, beats per min^C^	PDS+	38	119.8	3.8	111.8	3.2	109.9	4.8	116.9	3.4	115.5	4.2	114.7	3.4	110.2	5.6
	PDS−	38	111.4	3.5	108.1	3.1	108.0	4.6	110.7	3.1	117.6	4.9	102.4	3.3	97.1	4.3
Respiration, breaths per min	PDS+	38	42.2	1.1	42.9	1.1	44.4	1.1	48.1	1.1	26.4c	1.1	23.0a	1.1	21.3b	1.2
	PDS−	38	42.2	1.1	43.6	1.1	47.3	1.1	48.0	1.1	31.2	1.1	25.4a	1.1	22.9a	1.1
LPS, EU/mL	PDS+	38	10.31	1.62	17.03	1.43	7.34	1.63	16.72	1.43	9.55	1.67	19.00	1.52	11.56	1.68
	PDS−	38	7.78	1.62	13.46	1.49	7.48	2.06	9.85	1.42	4.57	2.74	22.77	1.41	27.03	2.76

Heart beats (beats per min), respiratory rate (respiration per min), expressed as least squares means, and concentration (EU/mL) of plasma LPS in blood from v. epigastrica cranials superficialis during 7 time slots (A–G, from −60 h antepartum until 36 h postpartum) in sows with (*n* = 38) and without (*n* = 38) postpartum dysgalactia syndrome (PDS+ and PDS−, respectively). An overall difference between PDS+ and PDS− sows are indicated by uppercase letters (heart rate): ^C^*P* < 0.01. Difference between time period A and period B–G is indicated by lowercase letters (respiration): ^a^*P* < 0.0001, ^b^*P* < 0.001, ^c^*P* < 0.01, ^d^*P* < 0.05.

**Table 3.** Clinical examination of mammary glands and capillary refill times

Variables	Group	Period	OR	LSMEANS	LSMEANS	LSMEANS	*P* value
				%	Lower %	Upper %	All over*
Mammary edema, yes/no	PDS−		1	8.78	6.05	12.58	
	PDS+		1.97	15.96	10.86	22.84	<0.05
		A (−60 to −36 h)	1	11.00	4.07	26.47	
		B (−36 to −24 h)	1.39	9.50	6.38	13.91	
		C (−24 to −12 h)	1.75	12.77	8.32	19.10	
		D (−12 to 0 h)	1.14	15.49	7.60	28.99	
		E (0 to 12 h)	1.26	10.65	6.60	16.74	
		F (12 to 24 h)	1.43	11.68	5.46	23.23	
		G (24 to 36 h)	1.18	13.08	7.84	21.03	0.68
Hard glands, yes/no	PDS−		1	3.18	1.96	5.12	
	PDS+		1.23	2.61	1.53	4.42	0.61
		A (−60 to −36 h)	1	4.67	1.72	12.10	
		B (−36 to −24 h)	2.67	0.92	0.35	2.42	
		C (−24 to −12 h)	2.53	2.42	1.17	4.93	
		D (−12 to 0 h)	2.43	2.29	0.56	8.88	
		E (0 to 12 h)	3.20	2.21	1.01	4.76	
		F (12 to 24 h)	12.24	2.89	1.41	5.85	
		G (24 to 36 h)	5.28	10.19	5.92	16.99	0.07
Prolonged capillary refill time in the glandular skin, >4 s	PDS−		1	40.71	31.69	50.40	
	PDS+		0.76	34.39	24.53	45.80	0.38
		A (−60 to −36 h)	1	56.13	41.98	69.36	
		B (−36 to −24 h)	0.82	51.24	39.38	62.97	
		C (−24 to −12 h)	0.37	32.21	17.83	50.99	
		D (−12 to 0 h)	0.58	42.61	31.53	54.48	
		E (0 to 12 h)	0.37	32.18	18.08	50.51	
		F (12 to 24 h)	0.30	27.98	17.64	41.33	
		G (24 to 36 h)	0.25	24.22	10.56	46.39	0.06
Prolonged capillary refill time in the vulvar mucosa, >2 s	PDS−		1	91.12	81.83	95.89	
	PDS+		0.22	69.48	53.43	81.87	<0.01
		A (−60 to −36 h)	1	89.27	77.16	95.35	
		B (−36 to −24 h)	0.74	86.05	75.72	92.43	
		C (−24 to −12 h)	0.58	82.83	65.75	92.38	
		D (−12 to 0 h)	0.95	88.77	78.43	94.50	
		E (0 to 12 h)	0.42	77.88	60.38	89.05	
		F (12 to 24 h)	0.47	79.75	67.09	88.38	
		G (24 to 36 h)	0.27	69.38	45.50	86.01	0.18

Least squares means is given for occurrence of mammary edema, hard glands, and capillary refill times of the glandular skin and vulvar mucosa during 7 time slots (A–G, from −60 h antepartum until 36 h postpartum) in sows with (*n* = 38) and without (*n* = 38) postpartum dysgalactia syndrome (PDS+ and PDS−, respectively).

*No interaction between GROUP and TIME was found for mammary edema (*P* = 0.64), prolonged capillary refill time in the glandular skin (*P* = 0.09), and prolonged capillary refill time in the vulvar mucosa (*P* = 0.21).

### Digestive Tract and Nutrition

Feces scores were not significantly different between the groups (*P* = 0.47; data not shown), but significant changes occurred in all sows at 0 to 36 h p.p. (*P* < 0.0001; data not shown) relative to the baseline. The changes were characterized by the transition from “normal feces” (score 2 or 3) to “dry” or an “absence of feces” (score 0 or 1). After farrowing, it was necessary to perform rectal sampling to obtain feces for scoring. This was required significantly more often compared with the baseline (*P* < 0.0001; data not shown), but did not differ between PDS+ and PDS− sows (*P* = 0.47; data not shown). Melena was not observed.

**Figure 1.**
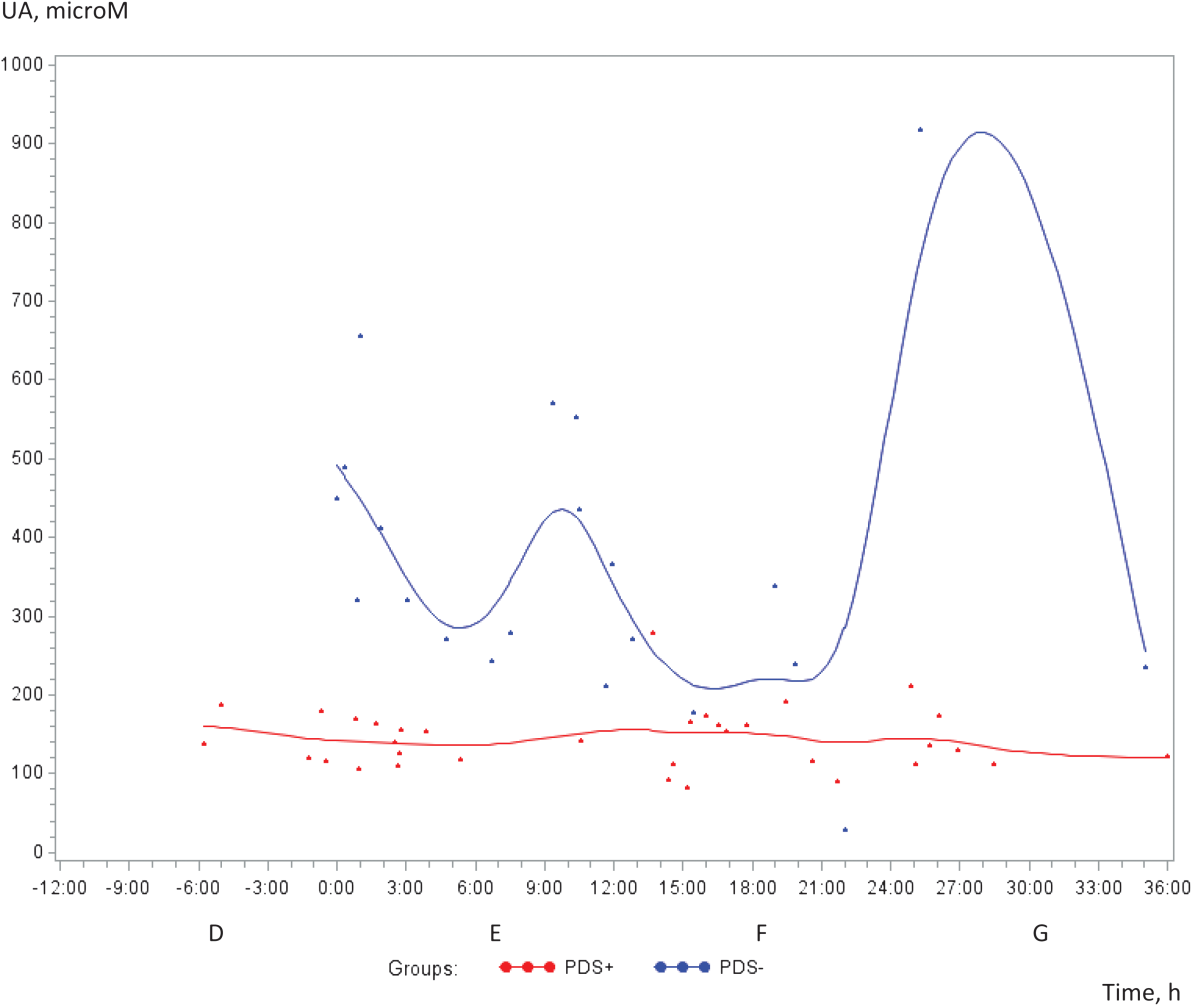
Concentration of uric acid (UA) (microM) in milk from 38 sows with postpartum dysgalactiae (PDS+, red line) and 38 healthy sows (PDS−, blue line) from −6 h antepartum until 36 h postpartum.

**Figure 2.**
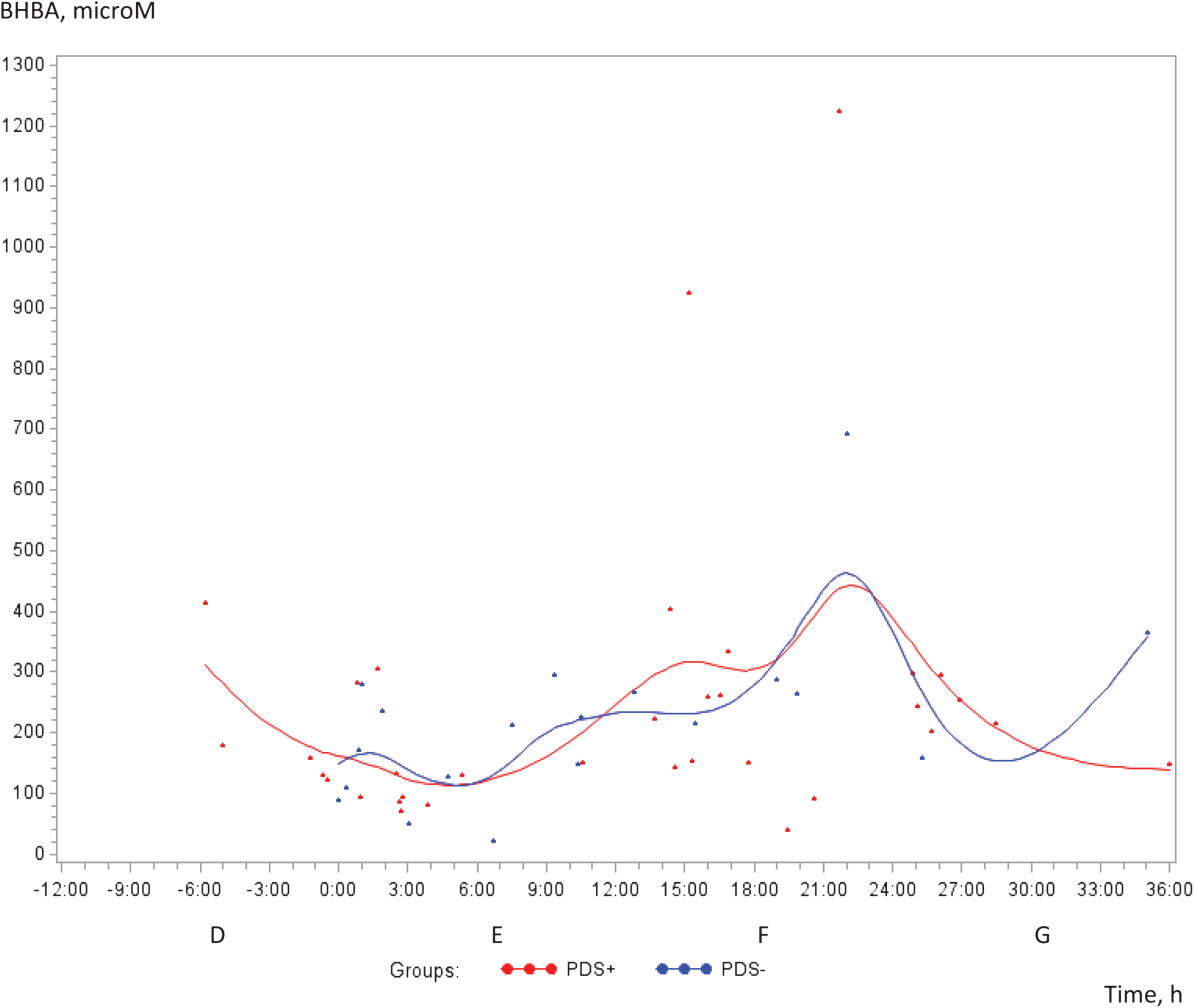
Concentrations of β-hydroxybutyrate acid (BHBA) (microM) in milk from 38 sows with postpartum dysgalactiae (PDS+, red line) and 38 healthy sows (PDS−, blue line) from −6 h antepartum until 36 h postpartum.

Concentrations of UA were significantly greater in PDS− sows than PDS+ sows (*P* < 0.0001), but no changes were demonstrated over time (*P* = 0.77; Fig. 1). β-Hydroxybutyrate acid increased over time (*P* < 0.05) with no difference between the groups (*P* = 0.92; Fig. 2). Free glucose, isoC and urea did not change over time (*P* = 0.33; *P* = 0.82; *P* = 0.95, respectively) and no differences between the groups appeared (*P* = 0.86; *P* = 0.73; *P* = 0.93, respectively; data not shown).

### Mammary Glands

Mammary edema was more severe in PDS+ sows than in PDS− sows (*P <* 0.05) in all time periods, but no change was observed over time (*P =* 0.68; Table 3). Capillary refill time of glandular skin and the occurrence of hard glands did not differ between the groups (*P =* 0.38; *P =* 0.61, respectively) or change over time (*P =* 0.06; *P =* 0.07, respectively; Table 3).

Substantial bacterial infections appeared infrequently and with no differences between the groups. The bacteria detected were coliforms (3 PDS+, 1 PDS−), *Staphylococcus aureus* (2 PDS+, 1 PDS−), and *Staphylococcus hyicus* (1 PDS+, 1 PDS−).

**Figure 3.**
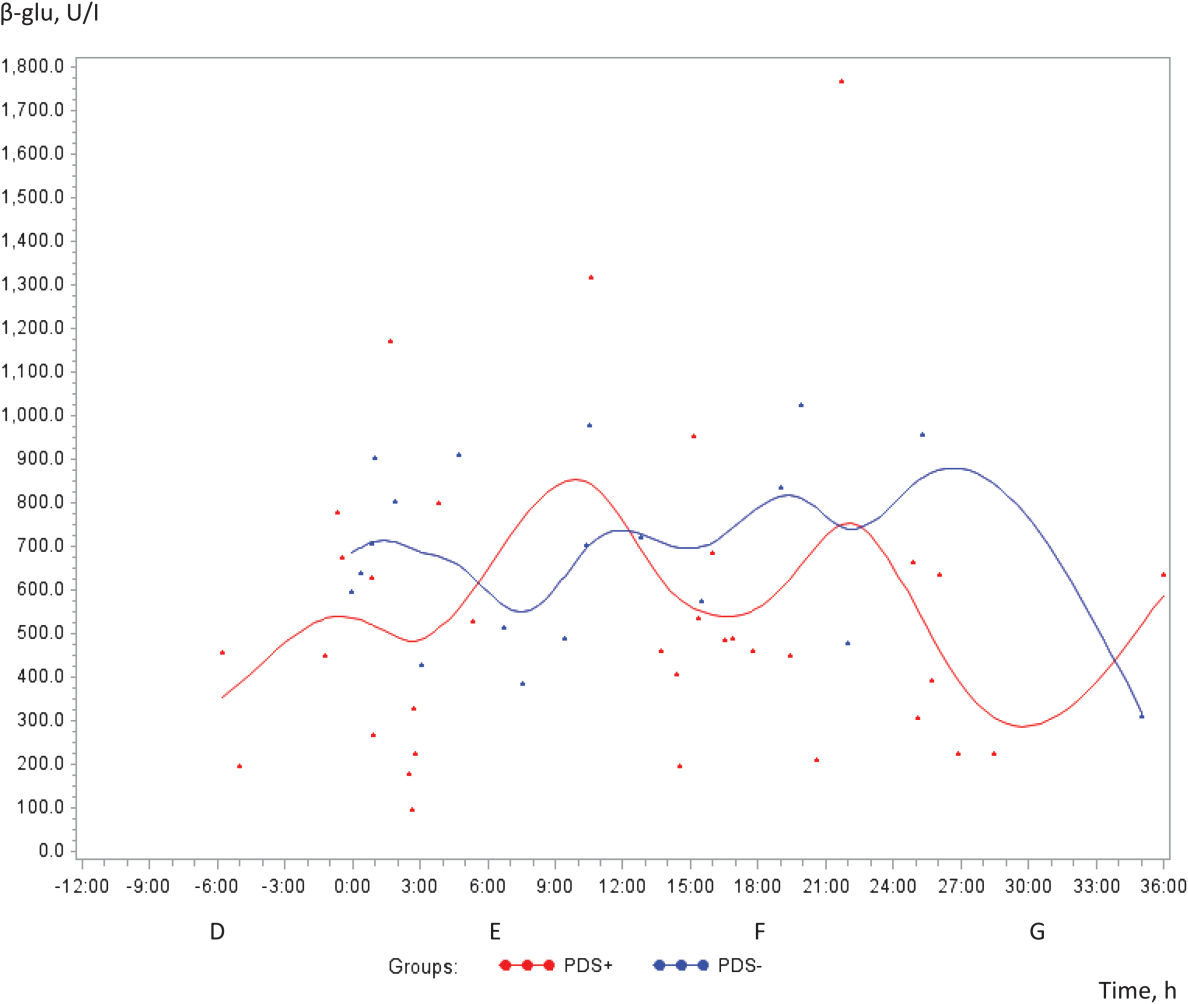
Concentrations of β-glucuronidase (β-glu) [nanomoles of product/min × liter (U/I)] in milk from 38 sows with postpartum dysgalactiae (PDS+, red color) and 38 healthy sows (PDS−, blue color) from −6 h antepartum until 36 h postpartum.

The healthy PDS− sows had significantly greater concentrations of β-glu than PDS+ sows (*P* < 0.05), but no change in concentration was demonstrated over time (*P* = 0.79; Fig. 3). Compared with the baseline, LDH and NAGase concentrations in milk did not change significantly over time (*P* = 0.11; *P* = 0.65, respectively), and there were no differences between PDS+ and PDS− sows (*P* = 0.85; *P* = 0.21, respectively; data not shown).

### Milk Vein LPS

Concentrations of LPS in milk vein blood did not change over time, nor did they differ between groups (Table 2). No correlations were demonstrated between concentrations of LPS and redness (*P* = 0.24), warmth (*P* = 0.07), hardness (*P* = 0.66), and edema (*P* = 0.54) in the 6 most anterior glands. Over time, the 6 most anterior glands became significantly redder (*P* < 0.0001), warmer (*P* < 0.0001), and harder (*P* < 0.05) relative to the baseline in all sows (Table 4).

**Table 4.** The associations between lipopolysaccharide (LPS) concentrations and mammary clinical variables

		Confidence interval			
Appearance of the 6 most anterior glands	Variables with potential effect on glands	OR	Lower	Upper	*P-*value
Red glands, yes/no	LPS, EU/mL	1			
		1.18	0.90	1.55	0.24
	A (−60 to −36 h)	1			
	B (−36 to −24 h)	0.97	0.27	3.42	
	C (−24 to −12 h)	6.93	1.17	41.02	
	D (−12 to 0 h)	1.38	0.45	4.24	
	E (0 to 12 h)	15.95	2.64	96.27	
	F (12 to 24 h)	23.75	6.70	84.21	
	G (24 to 36 h)	114.76	17.69	744.49	<0.0001
Warm glands, yes/no	LPS, EU/mL	1			
		0.76	0.56	1.02	0.07
	A (−60 to −36 h)	1			
	B (−36 to −24 h)	0.80	0.14	4.46	
	C (−24 to −12 h)	2.06	0.27	16.07	
	D (−12 to 0 h)	0.85	0.16	4.36	
	E (0 to 12 h)	23.54	3.32	166.62	
	F (12 to 24 h)	39.20	8.74	175.86	
	G (24 to 36 h)	103.36	14.57	733.33	<0.0001
Hard glands, yes/no	LPS, EU/mL	1			
		1.05	0.84	1.32	0.66
	A (−60 to −36 h)	1			
	B (−36 to −24 h)	5.07	0.80	32.13	
	C (−24 to −12 h)	3.52	0.42	29.45	
	D (−12 to 0 h)	2.29	0.36	14.59	
	E (0 to 12 h)	14.17	1.92	104.70	
	F (12 to 24 h)	6.71	1.15	39.28	
	G (24 to 36 h)	8.55	1.11	65.60	0.02
Edema, yes/no	LPS, EU/mL	1			
		0.94	0.78	1.14	0.55
	A (−60 to −36 h)	1			
	B (−36 to −24 h)	1.24	0.51	3.05	
	C (−24 to −12 h)	4.12	1.33	12.71	
	D (−12 to 0 h)	1.36	0.57	3.25	
	E (0 to 12 h)	2.10	0.62	7.09	
	F (12 to 24 h)	1.64	0.67	4.02	
	G (24 to 36 h)	1.70	0.50	5.80	0.25

Results of PROC GLIMMIX analysis of associations between LPS concentration (EU/mL) in v. epigastrica and mammary clinical variables (redness, warmth, hardness, and edema) in the 6 most anterior glands during 7 time slots (A–G, from −60 h antepartum until 36 h postpartum) in sows with (*n* = 38) and without (*n* = 38) postpartum dysgalactia syndrome (PDS+ and PDS−, respectively).

## DISCUSSION

### Mastitis

The inclusion criteria for diagnosing PDS in the current study were previously shown to distinguish between PDS+ sows that had an increased systemic inflammatory response and PDS− sows that had a significant reduced inflammatory response (Kaiser et al., 2018a). Interestingly, several of the results of the present study indicate that this inflammatory response is not associated with the development of mastitis. The gold standard for diagnosis of infectious mastitis is bacteriology, and a few samples, originating from both PDS+ and PDS− sows, showed evidence of bacterial infection. In addition, we saw no correlation between the LPS concentration in v. epigastrica and redness, warmth, hardness, and edema in the first 6 anterior glands. Moreover, no differences in LPS concentrations were detected between sows with and without PDS, indicating that increased inflammation was not induced by translocation of LPS from the udder to the bloodstream (Farmer et al., 2015). These results are in line with previous studies indicating that predisposing factors other than bacterial infections must be present for PDS to develop (Magnusson et al., 2001; Osterlundh et al., 2002; Gerjets et al., 2011; Kaiser et al. 2018b). However, Pejsak and Tarasiuk (1989) demonstrated a greater prevalence of LPS in sows with signs of coliform mastitis compared with healthy sows, indicating that LPS could be the cause of coliform mastitis in some cases. We only diagnosed a small number of cases of severe coliform mastitis, and the results therefore corroborate the suggestion that mastitis plays a secondary role (if any) in the pathogenesis of PDS (Armstrong et al., 1968). It may however still be possible that the number of sows with mastitis increased after the study was completed. Previous research indicates that *E. coli* is the dominant infection in milk from sows suffering from PDS with prevalence of 70% and 53.8%, respectively (Kemper et al., 2010; Angjelovski et al., 2016), but it may still be argued that bacterial infections occur secondary to a noninfectious factor, since the samples in these studies were collected relatively late (<12 h) after farrowing.

From a clinical perspective, we found that the diagnosis of mastitis was associated with a number of practical difficulties. The visual appearance of the udder was affected by factors like the sow’s position before the clinical examination (lying or standing), moisture on the floor, and a possible concurrent systemic inflammation. Milk accumulation and tension in the udder as well as piglet manipulation of the glands may also have affected results of the clinical examination, as these may elicit redness, warmth, and/or hardness.

It is likely that the difficulties faced in the assessment of udder health were simply related to the apparently low prevalence of mastitis. The finding that most often allowed us to classify sows as PDS+ was increased rectal temperature in combination with reduced appetite.

### Milk Markers

To our knowledge, UA in milk has not yet been investigated in sows. Uric acid is a degradation product from purines originating from DNA and RNA in cells. Because bacterial cells contain relatively more DNA than other constituents, UA has been used as an indicator of microbial cell degradation (Tas and Susenbeth, 2007). In cows, reduced UA concentrations in urine were related with reduced nitrogen flow to the duodenum and hence a reduced production of microbial protein in the rumen (Tas and Susenbeth, 2007). Recently, however, Larsen et al. (2016) found increased UA concentration in milk when cows were fed highly digestible feed versus a low-digestibility feed. The present finding of a reduced UA concentration in milk from PDS+ sows remains unclear, but against this background, it might be speculated that the intestinal function or health of PDS+ sows could be compromised.

The significance of the greater concentrations of β-glu in PDS− sows compared with PDS+ sows remains unclear. However, the measured values were well below the pathological concentration (15 U/mL) for mastitis in goats (Oliszewski et al., 2002) in both groups of sows, which supports the results of the bacteriological analyses, i.e., that most milk samples came from uninfected glands.

The significant increase in BHBA concentration in milk over time is linked to a small number of individuals (Fig. 2). A low incidence of ketosis in sows was previously described elsewhere (Hulten et al., 1993; Theil et al., 2013) so that it does not appear to be a significant problem in sows.

### Respiratory and Cardiovascular Systems

The cardiovascular system seemed to be compromised in PDS+ sows because the heart rate was increased in this group. The observed tachycardia could be caused by a sympathetic activation, as increased concentrations of chromogranin A (CgA) were demonstrated in PDS+ sows (Kaiser et al., 2018b). Chromogranin A was shown to regulate vascular homeostasis and the tension of the heart in humans (Helle and Corti, 2015), and increased concentrations are correlated with essential hypertension (Takiyyuddin et al., 1995). Furthermore, CgA interacts with endothelial cells and is associated with vascular leakage in mice (Ferrero et al., 2004). Because increased capillary wall permeability is central to edema (Cho and Atwood, 2002), it could be speculated that the mammary edema observed in PDS+ sows is a part of such a mechanism and is associated with the increased CgA concentrations. Prolonged capillary refill time is also a sign of circulatory disturbances (King et al., 2014), but the association with PDS is quite surprising as prolonged capillary refill time is primarily associated with severe disturbances (King et al., 2014), which were not present in the PDS+ sows.

The abrupt decreases in respiratory rate observed at the beginning of farrowing in the present study were previously been described (Hendrix et al., 1978). Respiration is partly regulated by n. vagus (Schelegle and Green, 2001; Chang et al., 2015), and the decline in respiratory frequency observed in healthy as well as PDS-affected sows could be explained by their general preference for lying down after farrowing (Špinka and Illmann, 2014), indicating a state of profound relaxation.

### Intestinal Motility

Constipation was previously linked to Mastitis, Metritis, Agalactia (MMA) (Hermansson et al., 1978; Jorsal, 1983), septicemia, and toxemia (Bosted et al., 1998). However, constipation appeared in most sows, and our results therefore suggest that the problem is not directly related to PDS. This is in line with previous studies showing no differences in the daily weight of feces between agalactic and clinically healthy sows (Nachreiner and Ginther, 1972). Feed intake close to farrowing was previously shown to be reduced in healthy sows and digesta transit time was increased, which may explain the tendency for constipation (Verstegen et al., 1998). The problem may be further exacerbated by increased intestinal water absorption at farrowing caused by a greater demand due to milk production (Mroz et al., 1995).

Although constipation seems to be common, it is possible that some individuals are more prone to the condition than others, e.g., from a disturbance in the gut microbiota causing inflammation. As reviewed by Agyekum and Nyachoti (2017), sufficient dietary fiber intake will result in a reduced digesta transit time and subsequently prevent opportunistic pathogens from colonizing the intestine. Providing high fiber diets during the periparturient period will reduce prolonged constipation in sows and promote piglet growth (Oliviero et al., 2009). An association was demonstrated between dysgalactia in sows and an increased occurrence of Gram-negative bacteria in the ileum (Morkoc et al., 1983). Absorption of LPS by the jejunum can also occur in some pigs (Vogelweid et al., 1981).

### Conclusions

We failed to confirm mastitis as a significant factor in the pathogenesis of PDS complex. Bacterial infections in the mammary gland occurred rarely in both PDS+ and PDS− sows, concentrations of LPS in plasma from v. epigastrica were not increased over time, nor were they any different between PDS+ and PDS− sows, and no correlation was found between LPS and clinical signs of inflammation in the udder. However, the cardiovascular system seems to be compromised in PDS+ sows, and this warrants further research.

## Supplementary Material

txz159_suppl_Supplementary_Appendix_1
