# Supplementary material for: Lack of evidence of mastitis as a causal factor for postpartum dysgalactia syndrome in sows
Source: Transl Anim Sci. 2019 Oct 11;4(1):250–63. doi: 10.1093/tas/txz159 (PMC6994082; doi:10.1093/tas/txz159)
Supplement: txz159_suppl_Supplementary_Appendix_1 [file txz159_suppl_supplementary_appendix_1.docx]

| **Clinical parameters** | | **Definition or description** |
| --- | --- | --- |
| **Rectal temperature^1^** | | |
|  | Fever | ≥ 39.5^o^C |
|  |  |  |
| **General demeanor** | | |
|  | 1. Alert | The sow seems fresh, awake and is interested in her surroundings. She eats her ration and is concerned about her piglets^†^ |
|  | 2. Depressed | The sow does not eat all her ration and seems uninterested in her piglets |
|  | 3. Non-responsive | The sow lies still. She is not interested in her surroundings and does not get up at feeding time^*^ |
|  | |  |
|  | Shivering | A rhythmic muscular trembling observed when sow is lying and relaxed; Yes/No^†^ |
|  | |  |
| **Cardiovascular system** | | |
|  | Heart rate | Beats per minute |
|  | |  |
|  | Capillary refill time in the vulvar mucosa | Normal when the mucosa returns to normal pink colour within 2 seconds after digital pressure to the mucosa |
|  | |  |
| **Respiratory system** | | |
|  | Respiratory rate | Breaths per minute |
|  | |  |
| **Skin and mucous membranes** | | |
|  | Skin colour | The colour on the back and side of the body is pink; Yes/No |
|  |  | The colour on the back and side of the body is pale/whitish; Yes/No^†^ |
|  |  | The colour on the back and side of the body is reddish; Yes/No^†^ |
|  |  |  |
|  | Ocular mucosa | The ocular mucosa membranes are pink; Yes/No |
|  |  | The ocular mucosa membranes are pale/whitish; Yes/No^†^ |
|  |  | The ocular mucosa membranes are dark red; Yes/No^†^ |
|  |  | The ocular mucosa membranes are pale/whitish yellowish; Yes/No^*^ |
|  |  | The ocular mucosa membranes are pale/whitish bluish/cyanotic; Yes/No^*^ |
|  |  | The ocular mucosa membranes have a vascular congestion appearance with clearly increased vessel contour; Yes/No^†^ |
|  |  |  |
|  | Vulvar mucosa | The vulvar mucosa membrane is pink; Yes/No |
|  |  | The vulvar mucosa membrane is pale/whitish; Yes/No^*^ |
|  |  | The vulvar mucosa membrane is dark red; Yes/No |
|  |  | The vulvar mucosa membrane is pale/whitish yellowish; Yes/No |
|  |  | The vulvar mucosa membrane is pale/whitish bluish/cyanotic; Yes/No |
|  |  | The vulvar mucosa membrane has a vascular congestion appearance with a clearly increased vessel contour; Yes/No^*^ |
|  |  |  |
| **Digestion and nutrition** | | |
|  | **Appetite** |  |
|  | 1. Normal | The trough is empty (less than a handful of feed remains) 30 min. after feeding |
|  | 2. Reduced | The trough contains feed residues 30 min. after feeding |
|  | 3. Ceased | The trough contains the entire ration 30 min. after feeding |
|  | |  |
|  | **Body condition^2^** |  |
|  | 1. Very thin | Ribs are distinct and clearly visible and can be felt easily by palpation^*^ |
|  | 2. Thin | The ribs are visibly covered but can be felt by a light press of hand |
|  | 3. Medium | The ribs are covered and cannot be appreciated upon inspection, but can be felt by a firm palpation |
|  | 4. Fat | The ribs are completely hidden and cannot be felt^*^ |
|  | |  |
|  | **Feces score^3^** |  |
|  | 0 | Absence of feces |
|  | 1 | Dry and pellet-shaped |
|  | 2 | Between dry and normal |
|  | 3 | Normal and soft, but solid and well-formed |
|  | 4 | Between normal and wet, still formed but not solid |
|  | 5 | Very wet feces, unformed and liquid |
|  |  |  |
|  | Feces assessment by rectal examination | Yes/No |
|  | Melena | Blackish grainy feces indicating gastric ulcer; Yes/No^*^ |
|  |  |  |
| **Mammary glands** | | |
|  | Normal tensed gland | No inflammation in the gland but firmness due to milk tension; Yes/No |
|  | Reduced milk production | The gland tissue is lax and assessed to be totally or partly unproductive; Yes/No |
|  | Warm gland | Increased gland skin temperature compared to the skin on the stomach; Yes/No |
|  | Red gland | The gland skin is red; Yes/No |
|  | Edema | The gland feels doughy and pressure leaves an indentation; Yes/No |
|  | Hard gland | Increased firmness; Yes/No |
|  | Non-functional gland | The gland has a chronic and healed lesion; Yes/No |
|  | Normal capillary refill time on gland no. 4 | Normal when the skin 3 cm above *Papilla mammae* returns to normal pink colour within 4 seconds of firm digital pressure; Yes/No |
|  |  |  |
| **Locomotory system** | | |
|  | 1 | The sow gets up easily without signs of pain. All four legs support the body |
|  | 2 | The sow gets up easily when she is fed, but continuously takes small restless steps^†^ |
|  | 3 | The sow only uses one limb slightly less to support the body weight^*^ |
|  | 4 | The sow does not use one limb to support the body weight^*^ |
|  | 5 | The sow refuses to get up due to obvious pain in the limb(s)^†^ |
|  |  |  |
| **Reproductive system** | | |
|  | Discharge | Vaginal discharge is observed; Yes/No |
|  | Plug of mucus | Observation of a gelatinous plug that normally seals the opening of the uterus and is released when the cervix softens; Yes/No |
|  | White lochial discharge | Lochial vaginal discharge related to the early *post-partum* period; Yes/No^†^ |
|  | Yellowish discharge | Purulent vaginal discharge; Yes/No^†^ |
|  | Reddish discharge | Purulent vaginal discharge with fresh blood; Yes/No^†^ |
|  | Brownish discharge | Purulent vaginal discharge with dark-brown blood; Yes/No^†^ |
|  |  |  |
|  | Vulvular trauma^2^ | A trauma including any kind of non-healed wounds in dermis or cutis; Yes/No |
|  | Ulcera^2^ | One or more ulcera including all kinds of non-healed wounds in dermis or cutis on the body; Yes/No^†^ |
|  | Abscesses ^2^ | One or more abscess on the body; Yes/No^†^ |
|  | Rectal prolapse | Yes/No |
|  | Vaginal prolapse | Yes/No |
|  |  |  |

^1^KRUUSE DIGI-TEMP Digital Thermometer, Jørgen Kruuse A/S, Langeskov, Denmark. Measurement temperature between 32-42^o^C. Accuracy of +/- 0.1^o^ C.

^2^These variables were only examined on day 1.

^3^The feces score was assessed according to Oliviero et al., 2009 (slightly simplified)

^*^Clinical findings with zero observations

^†^Clinical findings with few observations (≤ 5)
